# Prospective Evaluation of the Limb Reconstruction System as a Definitive Treatment for Compound Tibial Fractures: Radiological and ASAMI (Association for the Study and Application of the Method of Ilizarov) Functional Outcomes

**DOI:** 10.7759/cureus.94741

**Published:** 2025-10-16

**Authors:** Devendra Sharma, Ramsevak Sharma, Yogesh Singh Parihar

**Affiliations:** 1 Department of Orthopedics and Traumatology, Gajra Raja Medical College, Gwalior, IND

**Keywords:** asami score, compound tibial fracture, distraction osteogenesis, external fixation, limb reconstruction system (lrs)

## Abstract

Background

Open (compound) tibial fractures present a management challenge because of soft-tissue injury, contamination, and the risk of infection. The monolateral limb reconstruction system (LRS) is used as a definitive external fixation option that may permit stable fixation, wound access, and early weight-bearing.

Objectives

This prospective study aimed to evaluate the radiological and functional outcomes of the LRS used as a definitive fixation method in Gustilo-Anderson grade II-IIIB open tibial fractures, employing the ASAMI (Association for the Study and Application of the Method of Ilizarov) scoring system as a standardized assessment tool. The study also aimed to analyze the time to union and complication patterns to determine the feasibility and early functional recovery achievable with LRS as a single-stage management strategy in complex open tibial fractures.

Methods

This is a prospective single-center series of 30 consecutive adult patients (mean age = 32.7 years; 26 males) with open tibial shaft fractures treated definitively with the monolateral LRS. The Gustilo grade distribution was as follows: grade II = 14/30 (46.7%), grade IIIA = 10/30 (33.3%), and grade IIIB = 6/30 (20.0%). Primary outcomes were time to radiological union and ASAMI bone and functional scores at final follow-up. Secondary outcomes included complication rates. Minimum follow-up was 12 months. Continuous variables are presented as mean ± SD (range) and categorical variables as counts and percentages, with 95% confidence intervals.

Results

Thirty patients (mean age = 32.7 years; 26 males) were included. The mean time to radiological union was 24.6 weeks (range = 20-34). ASAMI bone results were excellent in 12/30 (40.0%; 95% CI: 22.7-59.4) patients, good in 11/30 (36.7%), fair in 5/30 (16.7%), and poor in 2/30 (6.7%) patients. ASAMI functional results were excellent in 8/30 (26.7%; 95% CI: 12.3-45.9) patients, good in 15/30 (50.0%), fair in 4/30 (13.3%), and poor in 3/30 (10.0%) patients. Complications included pin-tract infection in 6/30 (20.0%; 95% CI: 7.7-38.6) patients and joint stiffness in 4/30 (13.3%; 95% CI: 3.8-30.7) patients. There were no cases of non-union, implant failure, or deep infection (0/30; 95% CI: 0-11.6 for each).

Conclusion

In this prospective single-center series (n = 30), definitive fixation of open tibial shaft fractures with the LRS resulted in favorable radiological and functional outcomes, with acceptable complication rates. However, given the small sample size, single-arm design, and absence of a control group, these results should be interpreted cautiously. Larger, controlled studies are required to validate these findings and better define the comparative role of LRS in the management of compound tibial fractures.

## Introduction

Compound (open) fractures of the tibia are among the most frequently encountered injuries in orthopedic trauma, most commonly resulting from high-energy mechanisms such as road traffic accidents, falls from height, and industrial injuries. The tibia’s subcutaneous position along its anteromedial border renders it especially susceptible to soft-tissue compromise and infection when exposed through open fractures [1]. Recent studies estimate that open tibial fractures account for approximately 24% of all open fractures, with an incidence of three to four per 100,000 persons annually, most frequently affecting young adult males involved in road traffic accidents [2].

Open tibial fractures, particularly those classified as Gustilo-Anderson grade II and III, continue to pose major management challenges due to contamination, bone loss, devascularization, and the difficulty of achieving both stable skeletal fixation and adequate soft-tissue coverage. While internal fixation remains the gold standard for closed and low-grade (Gustilo-Anderson grade I) open tibial fractures, its use in higher-grade open fractures (grades II and III) is often limited by contamination, soft-tissue loss, and infection risk. In such cases, external fixation offers a safer and more practical alternative, providing stable fixation while allowing for wound management and soft-tissue reconstruction [3,4].

The limb reconstruction system (LRS) is a monolateral external fixator developed from Ilizarov principles, designed to address complex fractures, non-unions, and bone defects. It offers rigid stability, permits early weight-bearing, facilitates soft-tissue management, and enables postoperative dynamization, all of which contribute to faster rehabilitation and functional recovery [5]. The ASAMI (Association for the Study and Application of the Method of Ilizarov) scoring system provides an objective and standardized framework for evaluating both bone union and functional outcomes after external fixation [6].

This prospective study aims to evaluate the radiological and functional outcomes of the LRS as a definitive fixation method for compound fractures of the tibia using the ASAMI scoring system. While LRS has been extensively used as a temporary or staged fixation device, there remains limited prospective evidence assessing its efficacy and safety as a single definitive treatment modality in open tibial fractures. This study, therefore, seeks to address that gap and contribute to the existing body of literature on the management of complex tibial injuries [7].

Although several studies have reported satisfactory outcomes with the LRS in managing open tibial fractures, much of the earlier literature, including the foundational work by Campbell and Paley, focused primarily on biomechanical principles and early clinical applications rather than contemporary prospective outcome data [5,6]. More recent research has emphasized the ongoing challenges in treating Gustilo-Anderson grade II and III tibial fractures, particularly regarding infection prevention, bone union, and soft-tissue coverage [7,8]. However, most available studies remain retrospective, involve mixed cohorts of open and closed fractures, or evaluate LRS mainly as a temporary or staged fixation device rather than as a definitive treatment method [7-9].

The present prospective study aims to address this gap by evaluating both radiological and functional outcomes of the LRS when used as a definitive fixation modality for compound tibial fractures, assessed using the ASAMI scoring system. By focusing on a homogeneous cohort of open fractures (Gustilo-Anderson grades II-IIIB) and applying standardized assessment criteria, this study provides updated clinical evidence supporting the potential of LRS as a reliable, single-stage fixation option in complex tibial injuries, especially within resource-limited trauma settings [9].

## Materials and methods

This was a prospective observational study conducted in the Department of Orthopedics at Gajra Raja Medical College and J.A. Group of Hospitals, Gwalior. Ethical clearance was obtained from the Institutional Ethics Committee. Informed written consent was obtained from all participants prior to inclusion in the study.

This study was designed as a prospective observational series to evaluate the feasibility and outcomes of the LRS as a definitive fixation method for open tibial fractures. A total of 30 consecutive eligible patients who met the inclusion criteria during the study period were enrolled. No formal sample-size or power calculation was performed, as the primary intent was to generate preliminary prospective data for hypothesis formulation and to estimate effect sizes for planning larger comparative studies. Patients were selected from the emergency and outpatient departments and were followed up postoperatively for a minimum of 12 months.

Inclusion criteria

Inclusion criteria included age ≥18 years, compound (open) fracture of the tibial shaft classified as Gustilo-Anderson grade II or higher, willingness to undergo external fixation with LRS as a definitive treatment, and ability to adhere to scheduled follow-up visits.

Exclusion criteria

Exclusion criteria included Gustilo-Anderson grade I fractures, pathological fractures, patients with neurovascular injuries requiring repair, patients previously treated with internal fixation or alternative external fixation, and medical contraindications to surgery or anesthesia.

Initial management

All patients presenting with compound tibial fractures underwent initial resuscitation and wound care per Advanced Trauma Life Support (ATLS) protocols. Intravenous antibiotics were administered immediately on arrival, typically including a first-generation cephalosporin and aminoglycoside, with metronidazole added for contaminated wounds. Tetanus prophylaxis was ensured.

Surgical technique

All patients underwent thorough wound debridement under regional or general anesthesia as soon as their clinical condition permitted, preferably within six to eight hours of injury. Devitalized tissue, foreign material, and gross contaminants were meticulously removed. Fracture ends were freshened until punctate bleeding was observed, indicating viable bone.

The LRS was applied after provisional fracture alignment. Schanz pins of appropriate diameter were inserted percutaneously under fluoroscopic guidance, maintaining at least 2 cm distance from the fracture line. Pins were placed parallel to each other and perpendicular to the bone’s anatomical axis. The monolateral rail frame was then assembled and fixed securely to the pins to achieve stable fracture alignment in both coronal and sagittal planes.

Fracture reduction was confirmed radiologically, and any residual malalignment was corrected by adjusting the rail clamps before final tightening. The stability of the construct was reassessed through gentle manipulation. Pin sites were dressed with sterile gauze, and the wound was either closed primarily or managed with delayed closure, skin grafting, or flap coverage depending on soft-tissue condition.

Postoperative protocol

Postoperatively, patients were encouraged to begin early mobilization and partial weight-bearing as tolerated, depending on pain and stability. Pin-site care was performed daily, and radiographs were taken at regular intervals to assess alignment and callus formation. Dynamization was initiated once signs of union were evident, and the fixator was removed after achieving complete radiological and clinical union.

Follow-up and outcome assessment

Patients were followed up at monthly intervals for up to 12 months. During each visit, clinical evaluation included the following: assessment of pain, range of motion, limb alignment, and infection, and radiographic evaluation to assess union, callus formation, and alignment. Data were analyzed using IBM SPSS Statistics version 26 (IBM Corp., Armonk, NY). Continuous variables were expressed as mean ± standard deviation (range), and categorical variables as counts and percentages with 95% confidence intervals. Comparisons between fracture subgroups (Gustilo grade II vs. III) were made using the chi-square or Fisher’s exact test for categorical data and the Student’s t-test or Mann-Whitney U test for continuous data. A p-value <0.05 was considered statistically significant.

Functional and radiological outcomes were assessed using the ASAMI criteria [4], which include grading of bone results (union, infection, deformity, and limb length discrepancy) and functional results (pain, activity level, gait, and range of joint motion).

## Results

Patient demographics

Out of 30 patients enrolled, 26 (86.67%) were male and four (13.33%) were female, with a mean age of 32.7 years. The right leg was involved in 60% of cases. Road traffic accidents were the most common cause (80%), followed by falls (13.33%) and other trauma (6.67%). Due to the small and uneven distribution of patients across fracture grades (grade II: 14, IIIA: 10, IIIB: 6), no subgroup comparisons were performed. Descriptive outcomes are presented for the overall cohort. The detailed patient demographics and injury characteristics are mentioned in Table 1.

**Table 1:** Patient demographics and injury characteristics.

Parameter	Value
Number of patients	30
Male-to-female ratio	26:4
Mean age	32.7 years
Mechanism of injury	Road traffic accident (80%), fall (13.33%)
Side of injury	Right: 60%, left: 40%
Fracture location	Distal 1/3 (46.67%), middle 1/3 (36.67%), proximal 1/3 (16.66%)
Gustilo classification	Grade II: 14, grade IIIA: 10, grade IIIB: 6

Outcome assessment using the ASAMI criteria

The mean time to union was 24.6 weeks (range: 20-34 weeks). Radiological and functional outcomes were evaluated using the ASAMI criteria and discussed below in Tables 2, 3.

**Table 2:** ASAMI bone results. ASAMI: Association for the Study and Application of the Method of Ilizarov.

Outcome	Number of patients	Percentage (%)
Excellent	12	40%
Good	11	36.67%
Fair	5	16.67%
Poor	2	6.67%

**Table 3:** ASAMI functional results. ASAMI: Association for the Study and Application of the Method of Ilizarov.

Outcome	Number of patients	Percentage (%)
Excellent	8	26.67%
Good	15	50%
Fair	4	13.33%
Poor	3	10%

Complications

Complications were categorized using the Paley classification for external fixation-related events. The majority were minor and managed conservatively; no major complications, such as deep infection, non-union, or implant failure, were observed (Table 4).

**Table 4:** Complications following definitive fixation with the limb reconstruction system, categorized according to the Paley classification.

Complication type	Classification (Paley)	No. of patients (n = 30)	Percentage (%)	Management	Outcome
Pin-tract infection	Minor	6	20.0	Local care + oral antibiotics	Resolved
Joint stiffness	Minor	4	13.3	Physiotherapy	Improved
Pin loosening	Minor	2	6.7	Pin replacement	Resolved
Deep infection	Major	0	0	—	—
Non-union/implant failure	Major	0	0	—	—

## Discussion

Open fractures of the tibia, particularly Gustilo-Anderson grade II and III, are complex injuries due to their association with severe soft tissue damage, infection risk, and bone loss. These injuries typically result from high-energy trauma, such as road traffic accidents, the most common cause in our study [1]. The tibia’s subcutaneous location makes it especially prone to exposure and contamination, complicating management.

The LRS, a monolateral external fixator based on Ilizarov principles, is increasingly used in such cases due to its ability to maintain alignment, enable wound access, and allow early mobilization. In our study, LRS used as a definitive treatment yielded good to excellent bone results in 76.67% and functional outcomes in 76.67% of patients based on the ASAMI criteria [4]. The results of this small, single-arm series suggest that the LRS can provide satisfactory stability and functional outcomes in selected open tibial fractures; however, these findings should be interpreted as preliminary and require validation in larger, controlled studies. LRS’s ability to maintain periosteal blood supply and avoid deep soft tissue dissection contributes significantly to enhanced healing [5].

In our study, the average time to union was 24.6 weeks, comparable to previously published data on open tibial fractures managed with external fixation [2,3]. Pin-tract infections occurred in 20% of patients but were managed conservatively with local care and oral antibiotics, without progression to osteomyelitis, findings consistent with prior reports [6]. Joint stiffness was observed in 13.3% of patients, generally mild and attributed to delayed initiation of physiotherapy, which aligns with known functional challenges associated with external fixation. These comparable outcomes reinforce the potential clinical utility of the LRS in achieving satisfactory fracture healing and functional recovery; however, the absence of statistical comparisons between treatment modalities and the limited sample size necessitate cautious interpretation of these results.

Importantly, no patient experienced non-union, implant failure, or deep infection, and limb length discrepancy was not clinically significant. These outcomes suggest that when applied with proper technique and patient compliance, LRS is not only effective but also safe.

Given its affordability, modular design, and minimal invasiveness, LRS is especially valuable in resource-limited settings and plays a key role in damage control orthopedics. With appropriate patient selection and close follow-up, LRS serves as a reliable alternative to internal fixation methods in open tibial fractures [3].

Limitation

The absence of a control group is a major limitation, as it restricts direct comparison with other fixation methods such as intramedullary nailing or circular Ilizarov fixators. The present study was designed as a single-arm observational series to assess the feasibility and functional outcomes of LRS as a definitive fixation method in open tibial fractures. Future randomized or matched comparative studies are needed to determine relative efficacy and complication rates. The inclusion of Gustilo-Anderson grade II, IIIA, and IIIB fractures within a small cohort limits subgroup comparability and statistical inference. The present study serves as a pilot series; future multicenter or randomized studies with at least 20 patients per subgroup and an equivalent control arm (minimum total of 120 patients) are recommended to validate these findings.

The small sample size and absence of a formal power calculation limit the statistical generalizability of the findings. The study was conceived as an exploratory prospective series to generate data for subsequent larger-scale comparative trials.

Another limitation is the exclusive use of the ASAMI scoring system, which, although objective and standardized for external fixation outcomes, does not capture patient-reported quality-of-life data. Incorporating validated patient-reported outcome measures, such as the Short Form-36 (SF-36) or Lower Extremity Functional Scale (LEFS), in future research would provide a more comprehensive functional assessment.

## Conclusions

The present prospective single-center study evaluated the use of the LRS as a definitive fixation method for open tibial fractures. The technique demonstrated encouraging outcomes, with a mean union time of 24.6 weeks, satisfactory ASAMI bone and functional scores in the majority of patients, and manageable complication rates, including pin-tract infection in 20% and joint stiffness in 13.3%, all treated conservatively without progression to osteomyelitis. These findings indicate that LRS can provide stable fixation, allow early mobilization, and achieve reliable union even in complex open fractures.

However, the small sample size (n = 30), lack of a control group, and absence of advanced statistical analysis limit the generalizability and strength of these conclusions. The inclusion of mixed Gustilo-Anderson grades (II, IIIA, and IIIB) within a small cohort further restricts subgroup interpretation. Therefore, the results should be regarded as promising but preliminary, reflecting the feasibility and safety of LRS in selected cases rather than establishing its superiority over other fixation modalities. Future multicenter, randomized comparative studies with larger, stratified patient groups and incorporation of patient-reported outcome measures are recommended to validate these findings and better define the role of LRS in the definitive management of open tibial fractures.
